# Supplementary material for: Visualizing the gas-sensitive structure of the CuZn surface in methanol synthesis catalysis
Source: Nat Commun. 2024 May 8;15:3865. doi: 10.1038/s41467-024-48168-6 (PMC11079032; doi:10.1038/s41467-024-48168-6)
Supplement: Supplementary file 1 — Supplementary Information [file 41467_2024_48168_MOESM1_ESM.pdf]

Supplementary Information

Visualizing the Gas-sensitive Structure of the CuZn Surface in Methanol Synthesis Catalysis

Sigmund Jensen[1,†], Mathias H.R. Mammen[1,†], Martin Hedevang[1], Zheshen Li[2], Lutz Lammich[1,2], Jeppe V. Lauritsen[1]*

[1] *Interdisciplinary Nanoscience Center (iNANO), Aarhus University*

[2] *Department of Physics and Astronomy, Aarhus University*

**Email:* *jvang@inano.au.dk*

[†]*Shared first authorship*

Zn_{ad} island and CuZn alloy phase

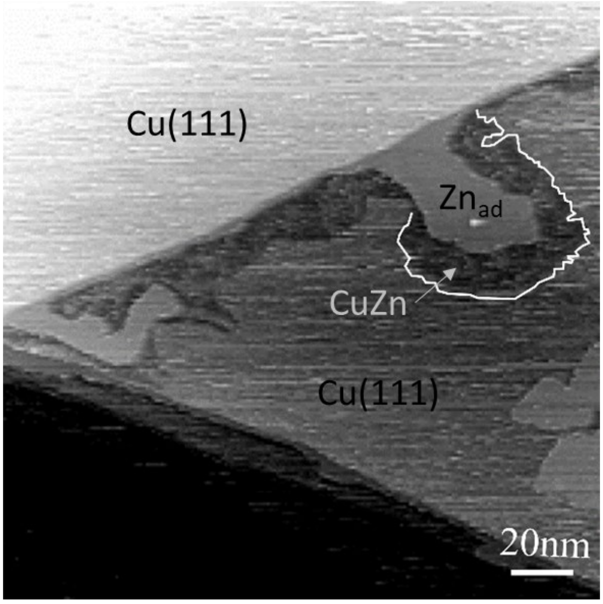

Supplementary Figure 1. Zn_{ad} islands created by direct Zn deposition are typically found to form adjacent to Cu(111) step edges. The white line reflects the boundary between CuZn (formed by Zn alloying from the edge of the Zn island at room temperature) and pristine Cu(111). The Zn_{ad} and Cu(111) step display an apparent height of ~1.9Å and ~2.1Å respectively. STM scanning conditions: U_t= 322 mV, I_t=0.47 nA.

Apparent height measurements

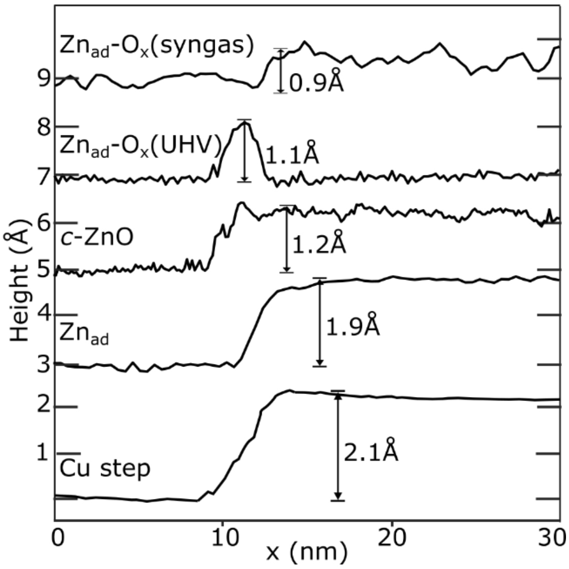

Supplementary Figure 2. Apparent height in STM. STM line profiles showing the apparent height of a Cu step along with Zn_{ad}, c-ZnO and Zn_{ad}-O_x structures. The apparent height of Zn_{ad}-O_x is measured both after the RT synthesis, formed by exposing the CuZn alloy to 2.1×10^{-9} mbar O_2 (UHV) and further measured for the Zn_{ad}-O_x structures forming during the 3 mbar $H_2/CO_2/CO$ exposure (syngas). The apparent heights of the different Zn structures were observed to depend little on the tunneling bias within the ± 1 V window, under which the majority of the imaging was performed. At an applied tunneling bias of 2.1 V the *c*-ZnO phase was found to display an apparent height of 2.5 Å showing that the imaging is bias dependent for the fully oxidized Zn phase. This is fully consistent with previous STM experiments on related ZnO[1] and ZnO/Cu(111) systems[2].

Table of measured apparent heights

UHV				
	Zn_{ad}	*c*-ZnO	Zn_{ad}-O_x	
Apparent Height (Å)	1.9 ± 0.1	1.2 ± 0.1	1.1 ± 0.2	
**In gas**				
		Zn islands (CO@1.5 mbar)	Zn_{ad}-O_x (H_2/CO_2/CO@3mbar)	Zn_{ad}-O_x (H_2/CO@3 mbar)
Apparent Height (Å)		0.9 ± 0.3	0.9 ± 0.3	0.9 ± 0.3
**After exposure**				
			Zn_{ad}-O_x (H_2/CO_2/CO)	Zn_{ad}-O_x (H_2/CO)
Apparent Height (Å)			0.9 ± 0.3	0.9 ± 0.3

Supplementary Table 1: Table of apparent heights measured for Zn_{ad}, c-ZnO, Zn_{ad}-O_x and Zn clusters, measured relative to Cu(111). The height analysis was performed for images acquired under UHV conditions, in gas and after near-ambient-pressure gas exposure. The average apparent height and the standard deviation in the apparent height are denoted for the different Zn phases. This is obtained by measuring the apparent height on ten different structures within each Zn-structure category, apart from the 3 mbar H_2/CO_2/CO and H_2/CO condition, where the challenging imaging conditions only allowed for five different height measurement in each case. All STM images used in the apparent height analysis were acquired with a positive bias within the 0.1-1V voltage range, under which tunneling conditions the apparent height does not show any strong dependency on the applied bias.

CuZn/Cu(111) imaged after CO_2 and H_2/CO_2 exposure

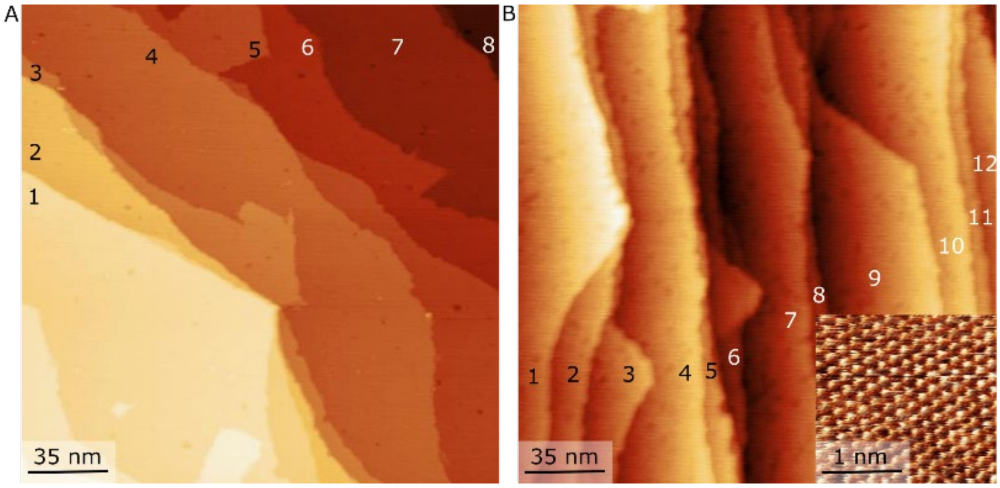

Supplementary Figure 3. (**A**) UHV-STM image acquired at 300 K after exposing the CuZn surface to 1.5 mbar CO_2 and heating the sample to 423 K. The surface is highly corrugated after exposure indicating that mass-transport has been operative under the mbar pressure and elevated temperature conditions. The numbers 1-8 denoting the number of terraces point out that the Cu surface was reshaping in gas. Zn_{ad}-O_x structures are not observed showing that the CuZn surface alloy is stable in presence of CO_2. (**B**) UHV-STM image acquired at 300 K after exposing the CuZn surface to a 3 mbar (2:1) H_2/CO_2 gas mixture and heating the sample to 423 K. Again, a highly corrugated surface is observed, indicated by the 12 surface terraces marked by the numbers 1-12. The inset in (**B**) further shows an atomically resolved STM image of the surface after H_2/CO_2 exposure. (**A**) STM scanning conditions: U_t= 361 mV, I_t=0.44 nA. (**B**) U_t= 153 mV, I_t=0.54 nA. for the large-scale image and U_t= 39 mV, I_t=0.89 nA for the inset.

NAP-STM of CuZn/Cu(111) in CO_2

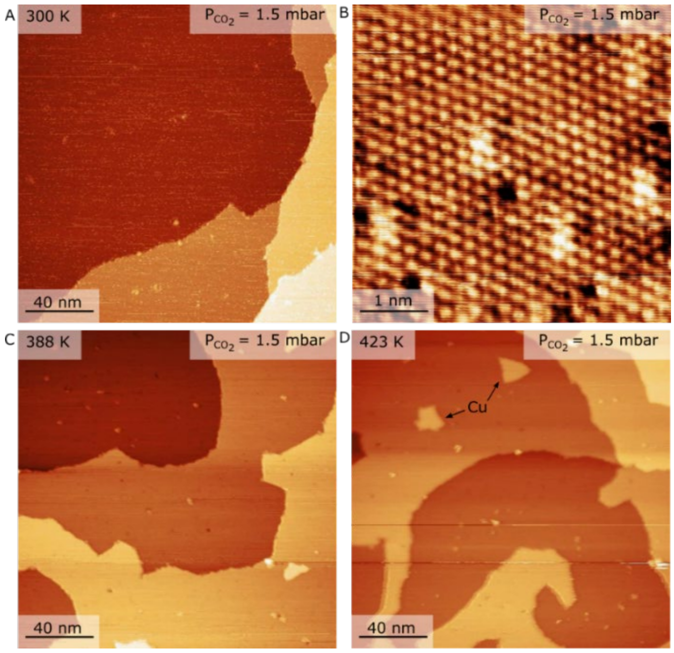

Supplementary Figure 4. NAP-STM images of CuZn in 1.5 mbar CO_2. (**A**) Overview STM image of the CuZn system acquired in a 1.5 mbar CO_2 gas environment. (**B**) On the smaller scale, Zn atoms in the Cu(111) matrix can be resolved. The Zn atoms are observed as bright protrusions exhibiting an apparent height of 0.3 ± 0.1 Å (**C**) The CuZn system imaged in 1.5 mbar CO_2 at 388 K. Two new surface phenomena are identified compared to the 300 K state being step edge reshaping and Cu island formation (apparent height 2.0 ± 0.1 Å). (**D**) Similar features are observed on the surface at 423 K, i.e., modified step edge structure and Cu islands (black arrows). Both the modified step edge structure and the Cu island formation strongly indicate that mass-transport of Cu is operative under the mbar pressure and elevated temperature conditions. It is challenging to separate the effect of CO_2 gas and temperature on the surface dynamics solely from NAP-STM imaging, as step edges on the Cu(111) surface are mobile even at 300 K[3]. We therefore ascribe the enhanced mobility to a combination of gas and temperature effects. STM scanning conditions for (**A-D**) respectively: U_t= 340 mV, I_t=0.43 nA. U_t= 90 mV, I_t=0.59 nA. U_t= 310 mV, I_t=0.52 nA. U_t= 270 mV, I_t=0.32 nA.

NAP-STM of CuZn/Cu(111) in H_2/CO

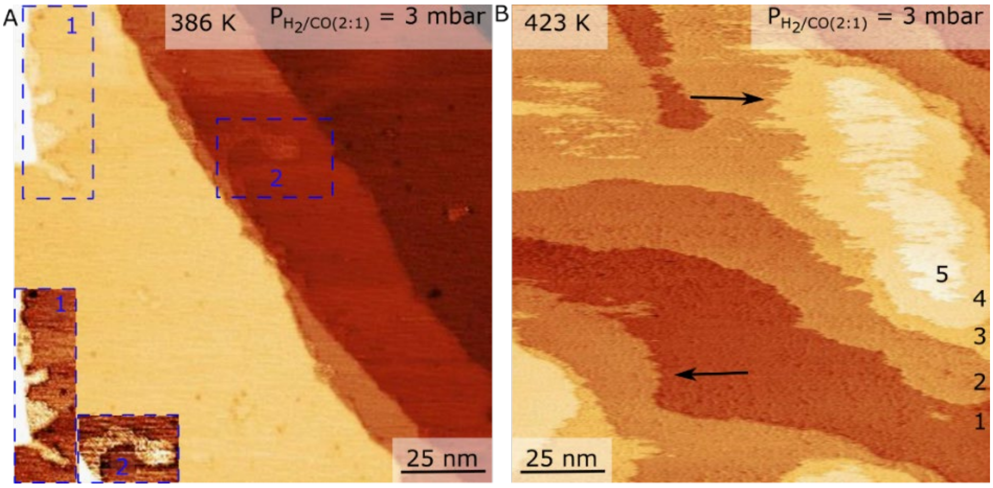

Supplementary Figure 5. NAP-STM of CuZn/Cu(111) in H_2/CO**.** No pronounced structural changes of the CuZn system were observed as the surface was imaged at 300 K in 3 mbar (2:1) H_2/CO (not shown). (**A**) In contrast, the NAP-STM image of the CuZn surface acquired at 386 K, reveals the formation of the Zn_{ad}-O_x monolayer phase (two highlighted in insets) exhibiting an apparent height of 0.9 ± 0.3 Å. The Zn_{ad}-O_x phase is predominantly observed adjacent to the step edges, which do not reshape significantly at this temperature under the CO hydrogenation conditions. (**B**) NAP-STM image showing the CuZn system at 423 K in the 3 mbar (2:1) H_2/CO gas mixture, the addition of H_2 clearly affects the mass-transport on the surface at this temperature point. The step edges appear smeared out indicating significant step edge mobility (marked by black arrow), likely due to an ongoing detachment of either Cu or Zn atoms (or both) emitted from the step site to the terrace of surface. The surface morphology is moreover characterized by being corrugated, evident from the high step density, as indicated by the numbers from 1-5, referring to the number of terraces. It was only possible to obtain a few images under these conditions signifying the on-surface dynamics under the mbar pressure and elevated temperature conditions. STM scanning conditions: U_t= 293 mV, I_t=0.41 nA and U_t= 318 mV, I_t=0.43 nA. for (**A**) and (**B**) respectively.

NAP-STM of CuZn/Cu(111) in CO

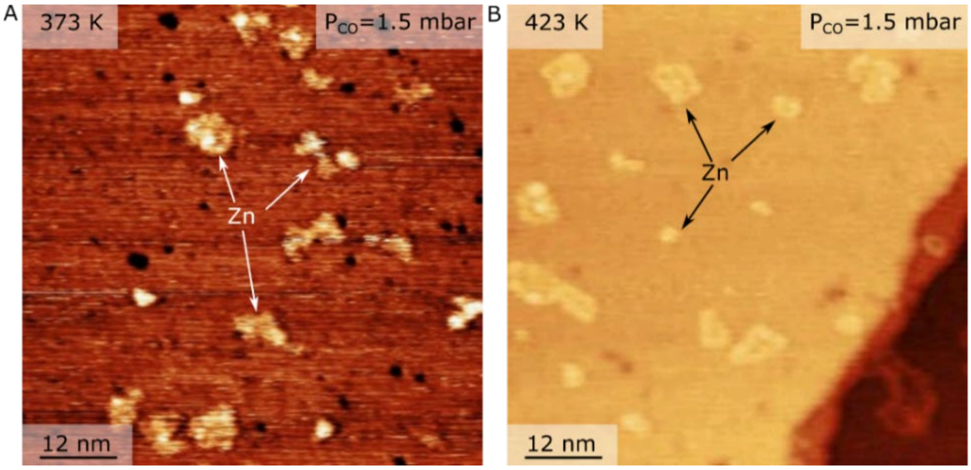

Supplementary Figure 6. NAP-STM of CuZn/Cu(111) in CO (**A**) NAP-STM image acquired at 373 K showing Zn islands (white arrow). The clusters exhibit different shapes, range in size from 20 Å to 85 Å (messured at the long axis) and displays an apparent height of 0.9 ± 0.3 Å, thereby appearing significantly lower than the adlayer Cu islands observed in the CO_2 and H_2/CO_2 gas mixture. (**B**) The Zn islands are again identified here at 423 K in the 1.5 mbar CO atmosphere (black arrow) in the NAP-STM image. The images under these conditions generally portray a surface covered in Zn islands. Moreover, the step edges of the surface appear less affected by CO compared to both the CO_2 gas and H_2/CO_2 gas mixtures at 423 K. The average size of the Zn islands of 49 Å ± 24 Å at 373 K and 64 Å ± 24 Å at 423 K (measured at the long axis of the islands) indicate that they are growing during the imaging series. STM scanning conditions for (**A**) and (**B**) respectively: U_t= 470 mV, I_t=0.53 nA and U_t= 336 mV, I_t=0.47 nA.

XPS after gas exposure in NAP-STM

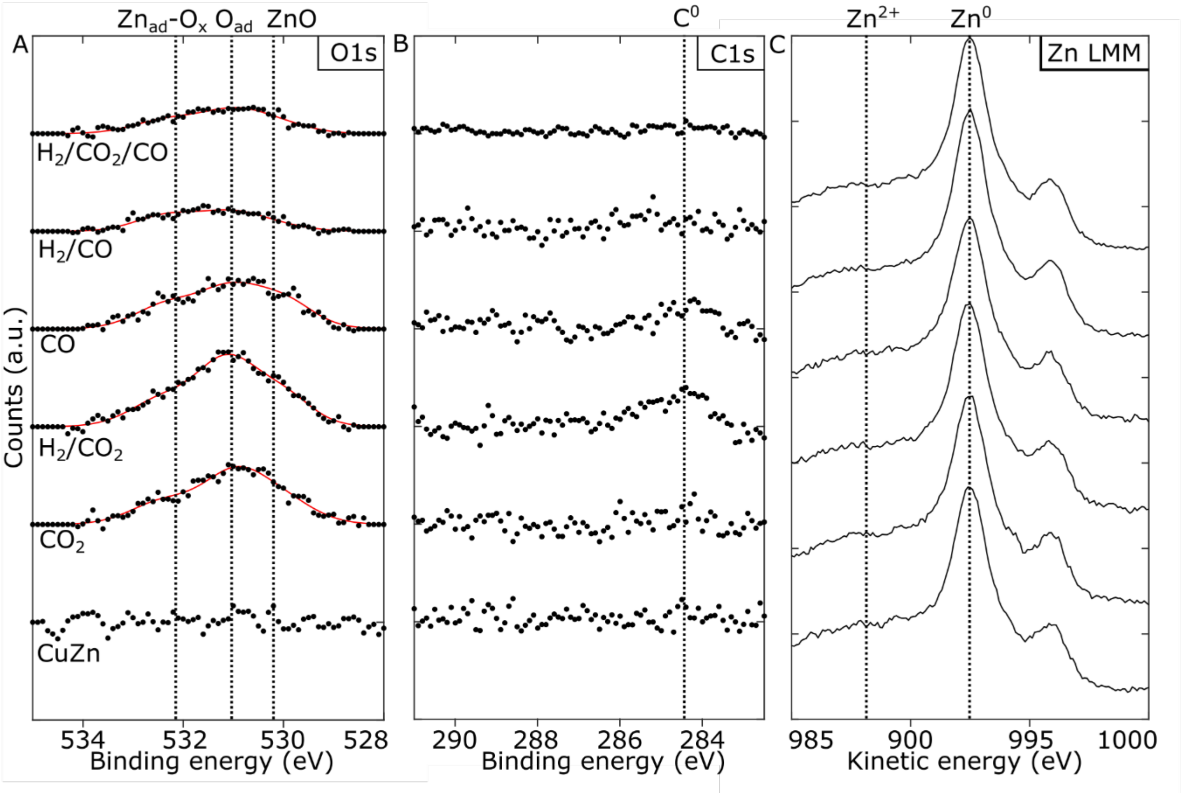

Supplementary Figure 7. Post-analysis XPS of the CuZn surface after mbar exposure of gas(es) recorded after transferring (through UHV) from the NAP-STM. The XPS (O*1s* and C*1s*) and Auger spectroscopy (Zn LMM) was performed in UHV under normal incidence with lab-source Al K_α radiation. Expected peak positions for Zn^0, $Zn^{2+}O$, chemisorbed O on Cu (O_{ad}) and adventitious C are indicated by the vertical lines. (**A**) O*1s* spectra after NAP-STM and transfer. The relatively broad O*1s* spectra complicate a spectral assignment, but they are likely explained by a combination of O species on Zn associated with the Zn_{ad}-O_x created during the NAP-STM experiment and adsorption of O species onto the Cu from the background gas during the XPS recording (several hours) (**B**) the C*1s* region is generally featureless, apart from a small signal located at the binding of 284.4 eV[6] (indicated by black line) corresponding to the core level position of adventitious carbon (C^0). Chemical signatures of surface species such as carbonate (CO_3) and formate (HCOO), relevant to the methanol synthesis reaction, are not observed[6]. Hence, it can further be ruled out that the CO_3 and HCOO species explain the broad O*1s* spectra. (**C**) The Zn LMM Auger peak displaying no overall difference between the Zn Auger peaks before and after the respective gas exposures, evidencing that the Zn remains metallic (Zn^0).

Zn_{ad} island dynamics

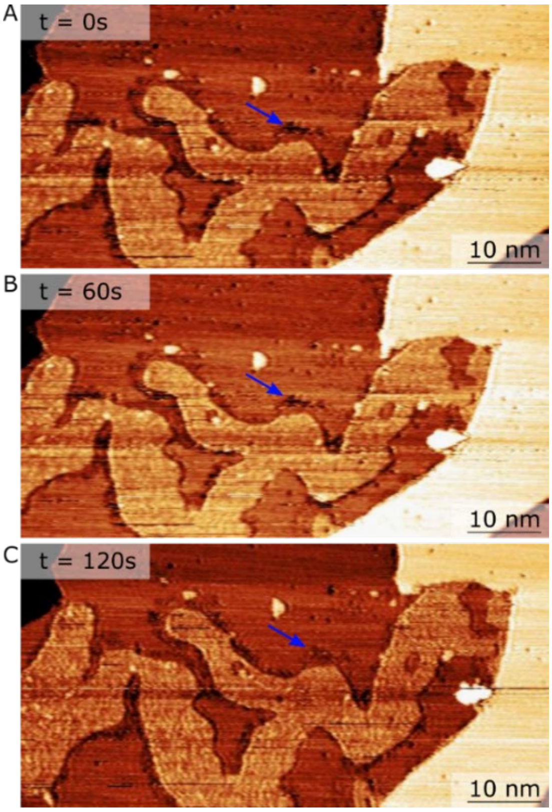

Supplementary Figure 8. (**A-C**) shows the evolution in the Zn_{ad}-O_x structure over a time period of 120s in 3 consecutive NAP-STM images. Generally, the Zn_{ad}-O_x structure remains stable under the mbar pressure and elevated temperature conditions, imposing that the gas induced dynamics have reach an equilibrium state. Small changes can though be observed in the darker appearing CuZn alloy brim region surrounding the structure (blue arrow), indicating some Zn atom exchange between the Zn_{ad}-O_x phase and the underlying Cu surface. STM scanning conditions : U_t= 153 mV, I_t=1.12 nA.

CuZn/Cu(111) exposed to 50 L CO

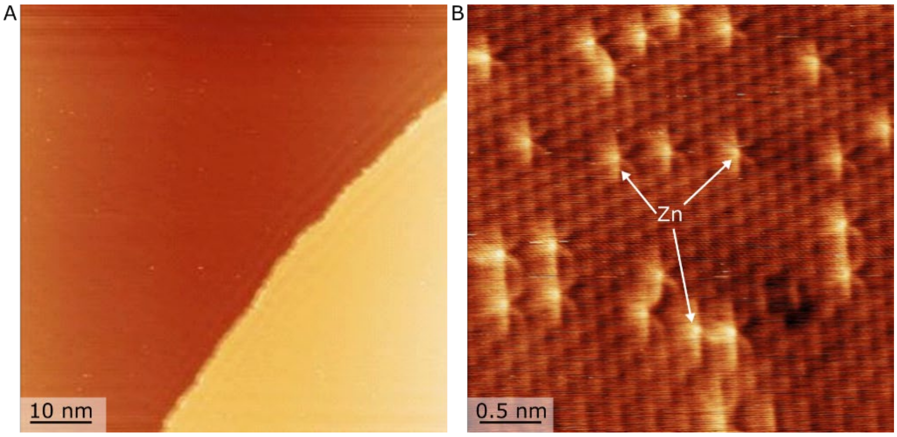

Supplementary Figure 9. (**A-B**) displays the CuZn alloy surface exposed to 50 L CO (1.1 × 10^{-7} mbar × 600 s). The STM images reveal that the CO-induced destabilization of the CuZn surface is not operative under the low-pressure conditions, in contrast to the elevated temperature and mbar pressure CO exposure. STM scanning conditions for (**A)** and (**B)** respectively: U_t= 70 mV, I_t=0.25 nA and U_t= 72 mV, I_t=0.36 nA.

Tip-sensitive imaging modes of Zn_{ad}-O_x

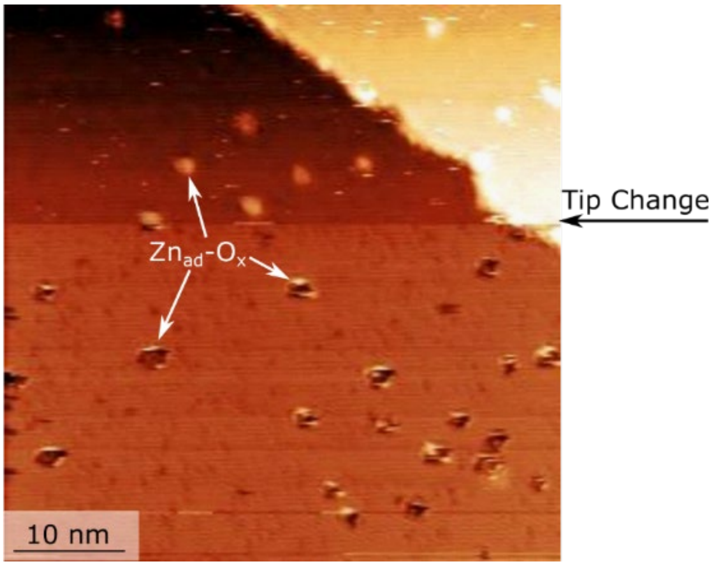

Supplementary Figure 10. Contrast of the Zn_{ad}-O_x phase due to STM tip changes. The STM image shows two distinct appearances of the Zn_{ad}-O_x phase, either imaged with a bright contrast (most common) in the top part of the image or dark contrast as seen in the lower part. The sudden tip change (black arrow) occurring during the image acquisition clearly illustrates the difference in contrast for the two distinct tip modes. STM imaging conditions: U_t = 379 mV, I_t = 0.34 nA.

Cu pit formation due to Zn extraction

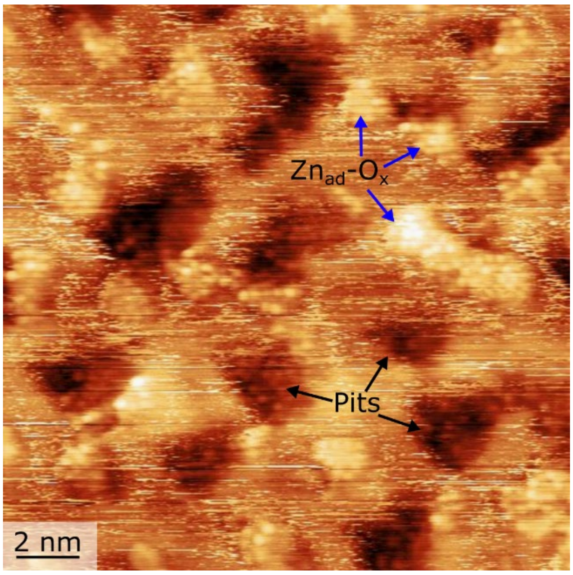

Supplementary Figure 11. STM images of Cu pit formation during O_2 exposure at room temperature. At later stages during O_2 exposure (here 40 L) to the CuZn system, pits (black arrows) in the Cu(111) progressively evolve on the surface together with the formation of Zn-O_x clusters (blue arrows). The apparent depth of the pits is 1.8 ± 0.2 Å corresponding to the height of a monoatomic step on the Cu(111). The pits are consistent with monolayer deep holes in the Cu(111) surface, formed as Zn atoms are abstracted from the Cu(111) matrix and due to mobility of Cu vacancies. STM scanning conditions: U_t= 90 mV, I_t=0.29 nA.

CuZn imaged after H_2/CO gas exposure

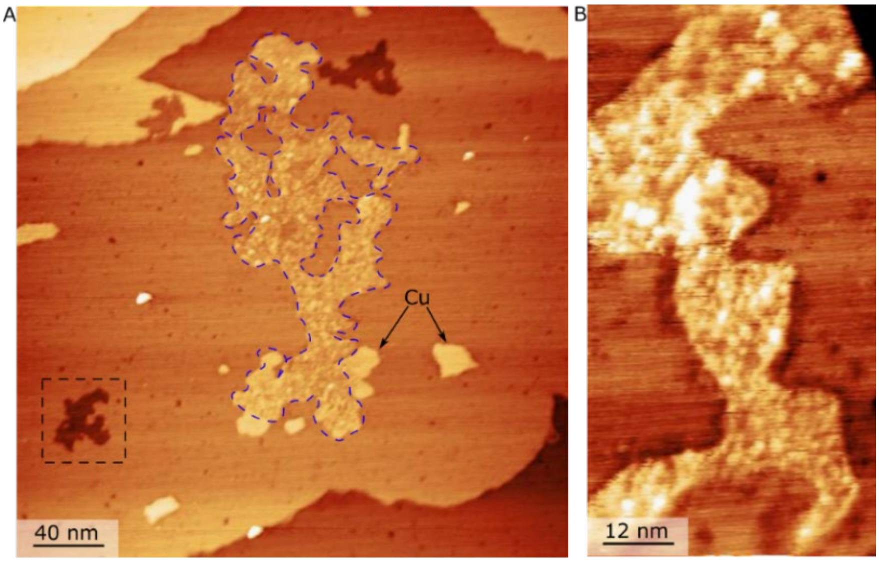

Supplementary Figure 12. Zn abstraction in after CO exposure **(A)** UHV-STM images acquired after the 3 mbar H_2/CO (2:1) gas exposure series. The surface is here covered in the Zn_{ad}-O_x phase (enclosed by blue line) resembling the structures observed *in-situ* during both the H_2/CO and H_2/CO_2/CO gas exposures. Cu_{ad} islands (black arrows) and monolayer deep irregular shaped holes (black square) are further seen. The Zn_{ad}-O_x phase, the Cu_{ad} islands and monolayer deep holes are identified from their apparent heights of 0.9 ± 0.3 Å, 2.0 ± 0.1 Å and an apparent depth of 2.0 ± 0.1 Å respectively. **(B)** UHV-STM image revealing the non-ordered adsorbate layer residing on the surface of the Zn_{ad}-O_x structure. The CuZn phase associated with the dark zone around the island perimeter is further surrounding the structure. It is noticeable that the surface of the Cu_{ad} islands is not covered in adsorbates. The holes are only observed after the H_2/CO and H_2/CO_2/CO gas exposures and are attributed to ensembles of agglomerated Cu vacancies, likely stemming from Zn extraction. STM scanning conditions for (A) and (B), respectively: U_t= 283 mV, I_t=0.51 nA and U_t= 322 mV, I_t=0.47 nA.

Supplementary References

1. Dumont, J. et al. ZnO(0001) surfaces probed by scanning tunneling spectroscopy: Evidence for an inhomogeneous electronic structure. *Appl. Phys. Lett.* **95**, 132102 (2009).

2. Liu, B. H. et al. Ultrathin Zn and ZnO films on Cu(111) as model catalysts. *Appl. Catal. A Gen.* **548**, 16–23 (2017).

3. Giesen, M. et al. Fast decay of adatom islands and mounds on Cu(111): A new effective channel for interlayer mass transport. *Phys. Rev. Lett.* **80**, 552–555 (1998).

4. Koitaya, T. et al. CO_2 Activation and Reaction on Zn-Deposited Cu Surfaces Studied by Ambient-Pressure X-ray Photoelectron Spectroscopy. *ACS Catal.* **9**, 4539–4550 (2019).

5. Nakamura, J. et al. Model studies of methanol synthesis on copper catalysts. *Stud. Surf. Sci. Catal.* **101**, 1389–1399 (1996).

6. Kahk, J. M. & Lischner, J. Core electron binding energies of adsorbates on Cu(111) from first-principles calculations. *Phys. Chem. Chem. Phys* **20**, 30403-30411 (2018).
